# A novel method for troubleshooting vascular injury during anatomic thoracoscopic pulmonary resection without conversion to thoracotomy

**DOI:** 10.1007/s00464-012-2475-1

**Published:** 2012-07-18

**Authors:** Jiandong Mei, Qiang Pu, Hu Liao, Lin Ma, Yunke Zhu, Lunxu Liu

**Affiliations:** Department of Thoracic Surgery, West China Hospital of Sichuan University, No. 37, Guoxue Alley, Chengdu, 610041 China

**Keywords:** Thoracoscopy/VATS, Lung cancer surgery, Lobectomy, Bleeding, Angiorrhaphy

## Abstract

**Background:**

Massive bleeding caused by vascular injury is considered the most troublesome and dangerous complication during video-assisted thoracoscopic surgery (VATS) pulmonary resection and is an important reason for emergency conversion to thoracotomy. The purpose of this paper was to show the suction-compressing angiorrhaphy technique (SCAT) for troubleshooting this problem without conversion.

**Methods:**

A total of 414 consecutive VATS anatomic pulmonary resections were performed between May 2006 and July 2011, among which 17 operations (4.11 %) encountered unexpected vascular injury. The procedure for troubleshooting vascular injury included bleeding control and angiorrhaphy. Bleeding was first controlled through side compression of the injured site with an endoscopic suction. Angiorrhaphy was then performed with running 5-0 Prolene suture using different procedures according to the size and location of the injuries, including direct suture upon suction compression, suture after substituting suction compression with clamping of the injured site, or suture after attaining proximal cross-clamping of the main pulmonary artery. Detailed information of these patients was carefully reviewed. The reasons for conversion to thoracotomy also were revealed.

**Results:**

Fifteen cases (15/17, 88.24 %) were successfully managed without conversion. Two cases of left main pulmonary artery injury were converted to thoracotomy due to difficulties in proximal cross-clamping of the injured vessel. Blood loss of the 17 patients ranged from 60–935 (median, 350) ml. Two patients were administered with allogeneic blood. The postoperative chest CT scan showed normal blood flow on the injured vessels. The total conversion rate was 2.66 % (11/414). The most common reason for conversion was hilar lymphadenopathy.

**Conclusions:**

The SCAT is an effective procedure for managing vascular injury during VATS anatomic pulmonary resection. In most cases, bleeding control and angiorrhaphy could be achieved using this method with acceptable blood loss, thereby avoiding emergency conversion to thoracotomy.

Video-assisted thoracoscopic surgery (VATS) for major pulmonary resection was first introduced in the early 1990s [1]. From then on, accumulated data have shown the advantages of this minimally invasive technique for the treatment of benign or malignant lung diseases. Two systemic reviews have summarized that the outcomes of VATS lobectomy for early stage non-small cell lung cancer (NSCLC) are comparable to those of open surgery [2, 3]. VATS lobectomy is now recommended as a reasonable and acceptable approach for patients with NSCLC if there are no anatomical or surgical contraindications [4].

The safety of conducting a VATS lobectomy is an important concern to surgeons, especially novices. This minimally invasive procedure may sometimes require emergency conversion to open surgery for a variety of reasons, such as technical difficulties, oncologic factors, hilar lymph node metastasis, uncontrollable bleeding, and completely fused fissure [3]. Serious bleeding is the most troublesome and dangerous condition of emergency conversion to thoracotomy, because the access is limited and is usually caused by unexpected vascular injury. Uncontrollable bleeding is one of the main reasons for conversion in some series of VATS lobectomies [5–9]. However, effective methods to manage this formidable problem without emergency conversion to thoracotomy are rarely reported in literature. In this study, we offer a novel method to manage vascular injury during VATS anatomic pulmonary resection with the help of an endoscopic suction. The procedure is called “suction-compressing angiorrhaphy technique” (SCAT). This study was approved by the institutional review board of our hospital.

## Materials and methods

### Patients

A total of 414 consecutive VATS anatomic pulmonary resections were performed by our group from May 2006 to July 2011. The hospital records of these patients were retrospectively reviewed. The cases of conversion from VATS to thoracotomy or patients who exhibited intraoperative vascular injury complications were identified. Seventeen patients (4.11 %) experienced unexpected vascular injury complication, among which 15 were successfully managed via the SCAT without conversion. The information on the characteristics of these patients, diagnosis, type of resection, reasons and site of vascular injury, operation time, intraoperative blood loss, blood transfusion, and postoperative complications was carefully reviewed. The reasons for conversion also were investigated. Informed consent of the operation was obtained from all patients before surgery.

### General procedures

Anesthesia was administered with a double-lumen endotracheal intubation. Patients considered as suitable candidates for VATS lobectomy were operated on using the single-direction lobectomy technique with single-lung ventilation, as previously described [10]. A 1-cm incision for the thoracoscope was made in the seventh intercostal space on the midaxillary line. A 3-cm utility incision was made on the anterior axillary line in the third intercostal space for upper and middle lobectomy and in the fourth intercostal space for lower lobectomy. A 2-cm assistant incision was made in the ninth intercostal space (between the posterior axillary line and scapular line). The vessels were dissected with a metal endoscopic suction with holes on the side of the tip and an electrocoagulation hook. Pulmonary veins and major branches of the pulmonary artery were cut down with endostaplers, and tiny branches of the artery were ligated with stitches or Hemolock clips. Patients with primary NSCLC underwent systemic lymph node dissection.

### Surgical techniques

**Fig. 1:**
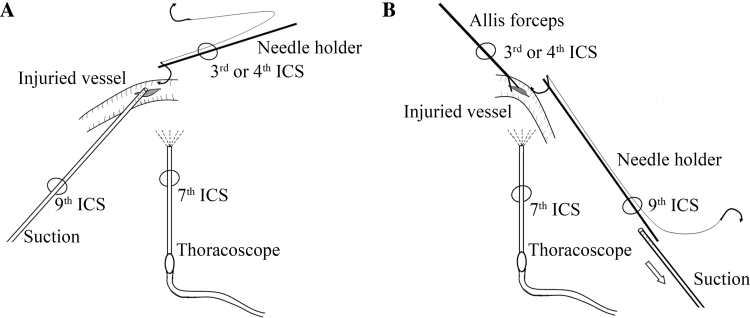
Typical placement of the ports and instruments. **A** Direct suture upon suction compression of the injured site. **B** Suture after substituting suction compression with clamping of the injured site. The suction was removed (*arrow*) after side clamping the wound with Allis forceps. *ICS* intercostal space

Vascular injury is usually an accidental situation during VATS lung surgeries. Thus, remaining calm and maintaining a clear thoracoscopic view when this troublesome crisis occurs is important. The procedure of SCAT could be divided into two steps. First, bleeding is controlled with an endoscopic suction, and then angiorrhaphy is performed. Placement of the instruments was slightly different between right and left side when performing SCAT (Fig. 1). No additional port was made when faced with vascular injury.

**Fig. 2:**
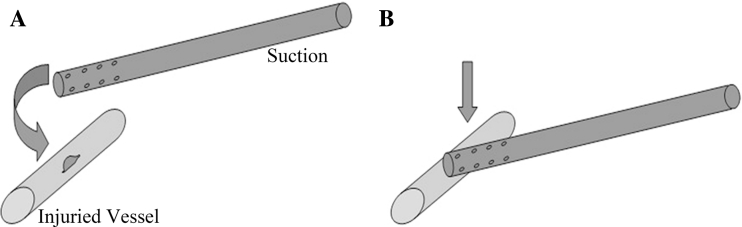
**A** Vascular injury. **B** Bleeding control via side compression of the injured site with the suction

An endoscopic suction with holes on the wall of the tip was used to control bleeding immediately after vascular injury. Bleeding was controlled through side compression of the injured site with the suction tip (the use of a finger to control bleeding in open surgery was imitated; Fig. 2). However, in cases where the suction is not in the thoracic cavity when bleeding complication occurs, suction should be inserted to reach the injured site to control bleeding as soon as possible. In this case, additional suction may be needed to clear the pooled clot when necessary.

The second step is to perform angiorrhaphy, which could be divided into three situations according to the size and location of the injury.

**Fig. 3:**
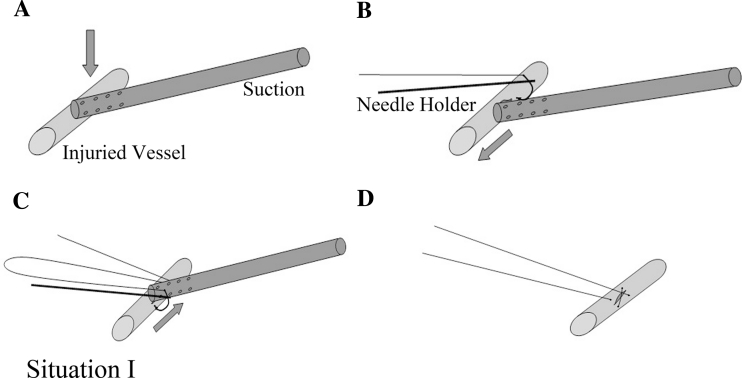
*Situation 1* Direct suture upon suction compression of the injured site. **A** Controlling bleeding with the suction. **B** and **C** Sewing the wound site by moving the suction in opposite directions. **D** Tightening the stitches
- Situation 2: Bleeding is usually difficult to control satisfactorily by using a suction tip during suturing when the vascular wound is larger than 5 mm but does not exceed one-third of the circumference of the vessel. The suction was substituted with long Allis tissue forceps, and the injured site was side clamped gently (Fig. 4A, B). Angiorrhaphy was then performed with a running 5-0 Prolene suture starting on one side of the Allis (Fig. 4C). The Allis was removed while tightening the stitches after sewing two sutures (Fig. 4D). An additional suture was made after removing the Allis. The wound was sewed again with the other needle of the same Prolene stitches from the same direction (Fig. 4E). The knot was finally formed by using a knot pusher (Fig. 4F).

- Situation 1: Angiorrhaphy is directly performed with a running 5-0 Prolene suture if the wound is shorter than 5 mm while the bleeding could be well controlled by the suction tip. The first suture was done on one side of the wound after slightly moving the suction tip to expose a part of the wound. The second suture was performed on the other side of the wound after moving the suction in the opposite direction, followed by tying the knot (Fig. 3). An additional suture may be needed in some cases.

**Fig. 4:**
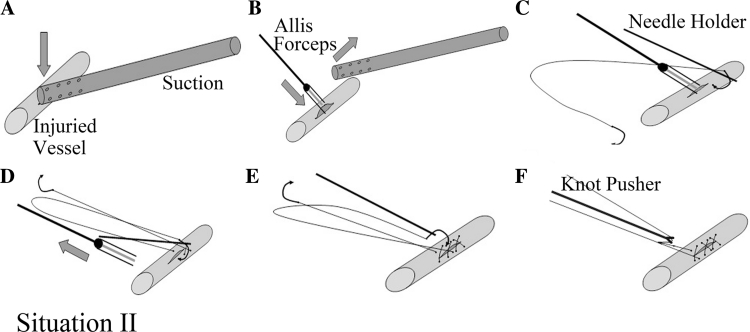
*Situation 2* Suture after substituting suction compression with clamping of the injured site. **A** Controlling bleeding with the suction. **B** Side clamping the wound with long Allis forceps and removing the suction. **C** Performing angiorrhaphy with running 5-0 Prolene suture on one side of the Allis. **D** Removing the Allis and making an additional suture. **E** and **F** Sewing the wound using the other needle of the same Prolene stitches and knotting
- Situation 3: Proximal main pulmonary artery should be dissected and clamped with an endoscopic atraumatic vascular clamp if the wound exceeds one third of the vascular circumference or when performing angiorrhaphy with an Allis in the chest is inconvenient. The suction used to control the bleeding was replaced by Allis tissue forceps, similar to the previously described, followed by dissection of the proximal artery to attain cross-clamping of the vessel. The Allis was removed after the proximal artery was clamped. Angiorrhaphy was then performed with a running 5-0 Prolene suture (Figs. 5, 6). Special attention is needed to avoid sewing contralateral wall of the injured vessel during angiorrhaphy.

**Fig. 5:**
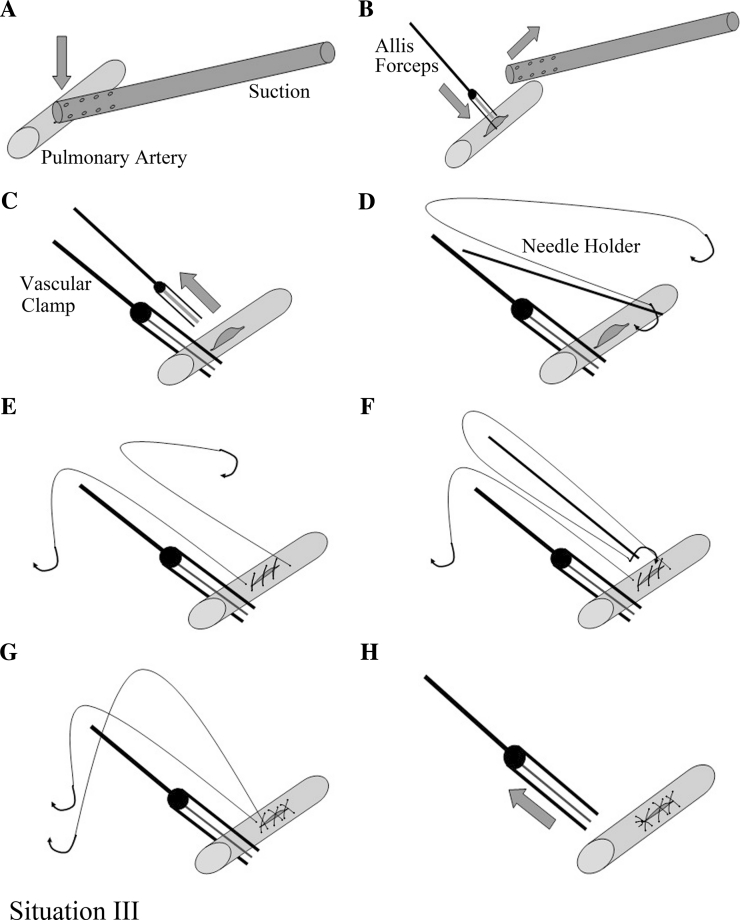
*Situation 3* Suture after substituting suction compression with proximal cross-clamping of the main pulmonary artery. **A** Controlling bleeding with the suction. **B** Side clamping the wound with long Allis forceps and removing the suction. **C** Clamping the proximal artery with an atraumatic vascular clamp and removing the Allis. **D** and **E** Performing angiorrhaphy with running 5-0 Prolene suture. **F** and **G** Sewing the wound using the other needle of the same Prolene stitches. **H** Removing the vascular clamp

**Fig. 6:**
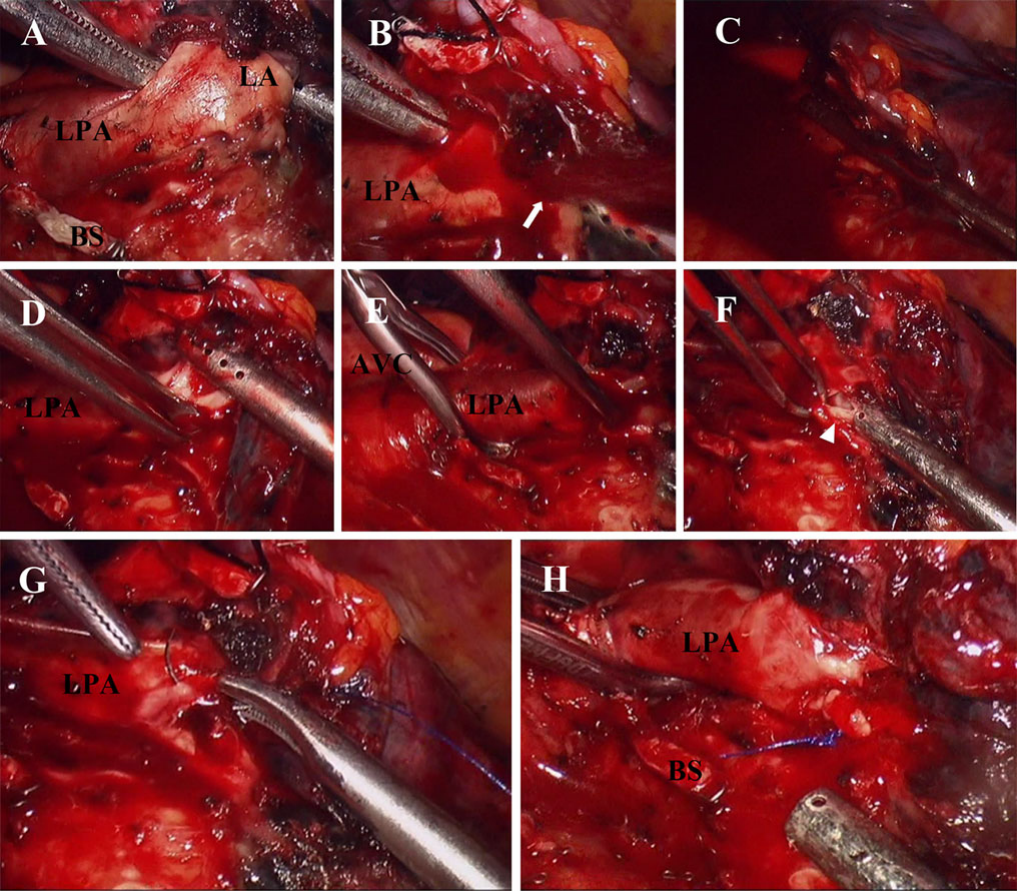
Demonstration of managing left pulmonary artery laceration using the method described in Fig. 5. **A** Blunt dissection of lingular artery with a long right angle clamp and suction. **B** Bleeding (*arrow*). **C** Controlling bleeding with the suction. **D** Clamping the laceration with Allis forceps and removing the suction. **E** Cross-clamping left main pulmonary artery with endoscopic atraumatic vascular clamp. **F** Reevaluating the laceration (*arrowhead*). **G** Sewing the laceration using running 5-0 Prolene suture. **H** Removal of the vascular clamp. *AVC* atraumatic vascular clamp, *BS* bronchial stump, *LA* lingular artery, *LPA* left pulmonary artery

### Postoperative management

Postoperative management was similar to our routine VATS lobectomy, and no anticoagulant agent was administered. The removal of the chest tube was performed without any air leakage, and the drainage was less than 200 ml for the previous 24 h. A contrast chest CT scan was performed during follow-up in patients who had intraoperative vascular injury complications.

## Results

In total, 414 patients underwent anatomic VATS pulmonary resections between May 2006 and July 2011, including 371 lobectomies, 19 bilobectomies, 15 segmentectomies, 5 concomitant segmentectomies and lobectomies, and 4 cases of VATS bronchial sleeve lobectomies. The diagnoses of these patients included 319 cases of primary lung cancers, 16 cases of lung metastasis, and 79 cases of benign pulmonary diseases. Eight patients were converted to standard posterolateral thoracotomy and three were converted to minithoracotomy with an 8 to 10-cm incision. The conversion rate was 2.66 % (11/414). The reasons for conversion are listed in Table 1.

**Table 1:** Reasons for conversion to thoracotomy

Reason	Number
Hilar lymphadenopathy	7
Chest wall invasion	1
Anatomic variation of pulmonary veins	1
Bleeding	2
Total	11 (2.66 %)

Seventeen patients (4.11 %, 17/414) experienced bleeding complication due to unexpected vascular injury. Fifteen of these patients (15/17, 88.24 %) were successfully managed using the SCAT without emergency conversion. The two cases of left main pulmonary artery injury were converted to thoracotomy due to the short route between the aortopulmonary ligament and the injured site and the difficulty to attain proximal cross-clamping of the injured vessel. The reasons of vascular injury included eight cases of sharp injury by the scissors, four cases of laceration by the endostapler, three cases of laceration during blunt dissection, one case of Hemolock clip loosening, and one case of coagulation hook leakage. The injured sites of these patients included 11 cases of pulmonary arteries, 3 cases of pulmonary veins, 2 cases of superior vena cava, and 1 case of anomalous artery of the sequestrated lobe. Detailed information of these patients is listed in Table 2. The blood loss of these 17 patients ranged from 60–935 (median, 350) ml. Two patients were administered allogeneic blood. The duration of the operations ranged from 85–260 (median, 180) min. The patients stayed in the hospital for 5–14 (median, 7) days after surgery. Six patients had postoperative complications; however, all of them recovered well with no mortalities. The postoperative chest CT scan showed normal blood flow in the injured vessels.

**Table 2:** Detailed information of patients who had vascular injuries

Pt	Sex	Age (year)	Diagnosis, p-TNM	Resection	Site of injury	Conversion	Blood loss (ml)	Transfusion	Time of op (min)	Postoperative complications
1	M	57	ADC, T_1a_N_0_M_0_	RLL	Superior vena cava	No	300	–	195	No
2	F	35	PS	RLL	Anomalous artery	No	500	–	190	Urinary tract infection
3	F	65	ADC, T_2a_N_0_M_0_	LLL	Lingular artery	No	495	–	210	No
4	M	63	M-rectal cancer	RML	Middle lobe artery	No	100	–	200	Sputum retention
5	M	62	M-lung SCC	LS	Lingular artery	No	570	–	130	Pneumonia
6	F	54	ADC, T_1a_N_0_M_0_	RLL	Superior vena cava	No	65	–	150	No
7	F	68	ADC, T_1a_N_0_M_0_	RML	Interlobar artery	No	300	–	190	No
8	F	41	Bronchiectasis	RML	Interlobar artery	No	350	–	95	No
9	F	72	ADC, T_1b_N_0_M_0_	LUL	Lingular artery	No	585	–	185	No
10	F	41	ADC, T_2a_N_1_M_0_	RUL	Anterior trunk	No	650	–	260	No
11	F	63	SCC, T_2a_N_2_M_0_	RLL	Anterior trunk	No	350	–	140	No
12	F	74	ADC, T_2a_N_0_M_0_	LUL	Main pulmonary artery	Yes	935	PRBC 2U	225	No
13	M	64	M-colon cancer	RLL	Common basal artery	No	150	–	120	No
14	M	69	ADC, T_1a_N_0_M_0_	LUL	Main pulmonary artery	Yes	810	PRBC 2U	185	Atrial fibrillation
15	F	53	ADC, T_2a_N_1_M_0_	RUL	Superior pulmonary vein	No	150	–	125	Cardiac insufficiency
16	M	56	PS	LLL	Lingular vein	No	60	–	85	No
17	M	75	SCC, T_2a_N_0_M_0_	RLL	Middle lobe vein	No	150	–	130	Arrhythmia

*ADC* adenocarcinoma, *LUL* left upper lobectomy, *LS* lingular segmentectomy, *LLL* left lower lobectomy, *M* metastatic, *Op* operation, *PRBC* packed red blood cells, *PS* pulmonary sequestration, *Pt* patient, *p-TNM* pathological tumor-node-metastasis staging, *RUL* right upper lobectomy, *RML* right middle lobectomy, *RLL* right lower lobectomy, *SCC* squamous cell carcinoma

## Discussion

VATS lobectomy for lung cancer was started in 2006 with a conversion rate of 2.66 % in our center. Sawada et al. [9] analyzed the reasons of conversion during VATS lobectomy and revealed that most of their conversions were caused by hilar lymphadenopathy, bleeding, and fused fissure. The single-direction method we used is a fissureless technique; thus, to date, no conversions have occurred for fused fissure in our center. The main reason for conversions in our center was dense hilar adhesion, usually caused by hilar lymphadenopathy.

The dense hilar adhesions of some patients may be attributed to their history of pulmonary tuberculosis or pneumoconiosis, and this contributes to high risk of vascular injury during surgery. Among the 17 patients with vascular injury, 8 had hilar adhesion due to lymphadenopathy, and the injury happened during sharp dissection with a pair of scissors. In addition, some unexpected events may cause intraoperative bleeding, such as loosening of the Hemolock clip or vascular laceration by the endostapler.

Unexpected bleeding is the most troublesome and dangerous condition during VATS lobectomy. Demmey et al. [11] provided solutions for bleeding during VATS lobectomy, such as using thrombostatic material, compression, or converting to open thoracotomy to obtain direct control. However, the use of thrombostatic material or mere compression is not enough to manage bleeding due to the injury of large vessels and angiorrhaphy is needed. Angiorrhaphy is difficult to perform without effectively controlling the bleeding and satisfactorily exposing the injured site. We designed a series of surgical skills for VATS bleeding control and angiorrhaphy during our practice to solve this problem without conversion to thoracotomy. This series of skills was called SCAT as introduced in this paper.

The endoscopic suction is used as an auxiliary instrument for dissecting the hilar structures and the lymph nodes. Therefore, suction is usually in the chest to facilitate the operation. This ensures the use of the suction to control bleeding immediately when accidental vascular injury is encountered. The suction can imitate the function of a finger in compressing the injured site of the vessel to stop bleeding. There are several advantages of using suction for bleeding control. First, due to the functionality of the suction, the device can rescue the injured vessel from pooled clot by clearing the blood and helping to expose the wound site accurately. Second, the device can keep on clearing any small amount of bleeding after controlling the major bleeding. These two advantages make the operation field relatively clear. Third, the suction offers better visualization of the wound site and gives space for further manipulation because of its elongated slender shape. Otherwise, directly compressing the injured site with gauze pieces also can stop the bleeding temporarily; however, the gauze may cover the surgical field and make the performance of further angiorrhaphy under thoracoscopy difficult. Therefore, conversion to thoracotomy would be the only choice during these cases. In some cases where the suction is not in the chest while bleeding complication is encountered, the suction should be inserted to the wound site to control the bleeding as soon as possible. However, the injured site may be quickly overwhelmed by the blood if the vascular wound is large. Therefore, additional suction is needed to clear the pooled clot.

Vascular bleeding is usually controllable at the outset but may turn into a disaster due to panic. Thus, remaining calm and managing the complication step-by-step are essential when faced with this complication. Performing angiorrhaphy directly in patients with vascular wounds not longer than 5 mm is relatively easy because bleeding could be well controlled by the suction tip. However, additional preparation should be done before performing angiorrhaphy for larger wounds. A pair of long Allis tissue forceps was used to side clamp the wound in our previous work because a more suitable instrument was absent. When using the Allis to clamp vascular wound, the ratchet should not be locked too tightly to avoid additional wound to the fragile vessels. The placement of the suction also may be adjusted between the ports after clamping the wound with an Allis.

Most vascular injuries were successfully managed via SCAT. However, two patients were converted to thoracotomy for treatment of the injury on the left main pulmonary artery because of anatomic limitations in attaining proximal control of the artery. The SCAT also has some limitations for the management of vascular injuries; thus, emergency conversion may be needed in some situations. In this case series, we failed to perform angiorrhaphy under thoracoscopy in two cases because of the short route between the aortopulmonary ligament and the injured site, where a vascular clamp should be placed. The bleeding could be temporarily controlled via side compression with the suction during thoracotomy. In cases where massive bleeding occurs due to a large rupture on the great vessels, such as accidental cross-sectional vascular injury (which we have never encountered until now), emergency thoracotomy is the most sensible choice.

Blood loss is an important concern in patients with vascular injury. The injury of great vessels, such as pulmonary artery or superior vena cava, may cause extensive bleeding in a short period. In this case series, the blood loss ranged from 60 to 935 ml (median blood loss was 350 ml). The two patients with left main pulmonary artery injury had blood loss of 810 and 935 ml, respectively. Only these two patients were administered with allogeneic blood. The blood loss was relatively acceptable in most of these patients, which was due to prompt bleeding control after vascular injury.

Watanabe et al. [12] introduced the technique of preventive pulmonary artery clamping using 1-0 silk suture. The main pulmonary artery was divided following double ligation with silk suture before lobectomy. This process is a good precaution for expected bleeding and can be done in patients with risk factors, such as dense hilar adhesions or lymphadenopathy. However, this procedure is not easy to perform among every patient, because the procedure itself might increase the probability of uncontrollable vascular injury. Some other reported skills for clamping the pulmonary artery during VATS lung surgery are feasible for managing malignant invasion or calcified lymphadenopathy involving the artery or the branches [13–15]. Sufficient mobilization of the proximal and distal artery is needed, as described in these reports. However, when unexpected vascular injury occurs, achieving proximal and distal control of the injured vessels for pulmonary artery clamping is difficult without controlling the bleeding.

In conclusion, the SCAT is an effective procedure for managing vascular injury during VATS anatomic pulmonary resection. In most cases, bleeding control and angiorrhaphy could be achieved by using this method with acceptable blood loss. Emergency conversion to thoracotomy also could be avoided in most cases.
